# Non-diabetic elderly populations: the MHR as a protective factor against bone abnormalities

**DOI:** 10.3389/fendo.2024.1408467

**Published:** 2024-06-07

**Authors:** Xiang Li, Manli Yan, Jiali Ji, Zhuohao Ma

**Affiliations:** ^1^Department of Orthopedic, Guangdong, Provincial Hospital of Chinese Medicine, Guangzhou, Guangdong, China; ^2^Second Clinical Medical College, Guangzhou University of Traditional Chinese Medicine, Guangzhou, China

**Keywords:** osteoporosis, non-diabetic population, the elderly, MHR, protective factor

## Abstract

**Objectives:**

In China, osteoporosis has become a major health concern among elderly population, imposing significant burden on the country’s social and economic systems. The monocyte to high-density lipoprotein ratio (MHR) has been currently recommended as a novel marker of inflammation and oxidative stress associated with osteoporosis in type 2 diabetes mellitus (T2DM). However, its reliability in non-diabetic elderly populations remains unclear. The present study was to evaluate the association between MHR and osteoporosis in a non-diabetic elderly population.

**Methods:**

The clinical data of 240 non-diabetic elderly subjects (115 in the osteoporosis group and 125 in the normal bone group) were retrospectively analyzed and all statistical analyses were performed by using SPSS 26.0.

**Results:**

Differences in age, neutrophils, lymphocytes, monocytes, MHR, uric acid, creatinine, triglycerides,and high-density lipoprotein cholesterol were found to be statistically significant between the two groups. A binary logistic regression model was conducted by including age, MHR, UA and Cr as variables. The results showed that age was an independent risk factor and MHR was an independent protective factor for bone abnormality in the non-diabetic elderly population. The ROC analysis showed that the area under the curve for the predictive effect of MHR, age and their combined test on osteoporosis in non-diabetic elderly populations was 0.623, 0.728 and 0.761, respectively; the correlation analysis showed that MHR was positively correlated with lumbar and hip BMD, and negatively associated with femoral neck stress ratio, femoral intertrochanteric stress ratio, and femoral stem stress ratio, showing statistically significant differences (P<0.05).

**Conclusions:**

For the non-diabetic elderly population: the MHR is a protective factor against bone abnormalities and was significantly higher in the normal bone group than in the abnormal bone group.

## 1 Introduction

Osteoporosis (OP) is a systemic metabolic bone disease characterized by low bone mineral density and microarchitectural destruction of bone tissue, thereby increasing bone fragility and susceptibility to fracture. According to data published by National Health Commission of the People’s Republic of China in October 2018 (1), the prevalence of osteoporosis among people over 50 years old was 19.2%, with a prevalence of 32.1% among women and 6.0% among men respectively. Osteoporosis patients are more prone to fragility fractures. It is roughly estimated that 20% of elderly hip fracture patients die of various complications within 1 year while 50% of patients become disabled. Therefore, OP has become an important health concern in the elderly Chinese population, resulting in socio-economic pressures in China.

OP is closely linked to chronic inflammation and abnormalities of glycolipid metabolism. In recent years, it has been found that OP is associated with chronic inflammation, and the negative balance between bone resorption and bone formation is the main mechanism of osteoporosis. For the pathogenesis of osteoporosis in the elderly population (1): Cellular ageing and functional degradation with human aging are the main factors. In addition, the differentiation of monocytes to form osteoclasts increases significantly with aging. This accelerates the bone resorption response, resulting in bone loss and metabolic bone diseases (2). The relationship between osteoporosis and dyslipidemia, especially in HDL-C metabolism (3), is complex and varies among different subgroups (4).

Monocyte to HDL ratio (MHR), initially considered to be associated with peripheral arterial disease, is a novel inflammation and oxidative stress marker. As the research progressed, doctors discovered that (5) MHR is closely related to osteoporosis in elderly type 2 diabetic patients; After analyzing the clinical data of 619 postmenopausal patients with T2DM, Huang Rong (5) et al. claimed that MHR could be an ideal marker to reflect the imbalance of bone homeostasis caused by chronic inflammation in Chinese postmenopausal women with T2DM; Zhou, Fushan (6) et al. found that MHR was negatively correlated with the risk of bone mineral density, bone loss and osteoporosis in women in their study of 1,804 cases of people aged 50 years or above; The study focused on analyzing the value of MHR in different genders, without involve distinguishing between diabetic and non-diabetic populations. In diabetic and non-diabetic populations, there are relatively different chronic inflammatory states and glycolipid metabolic states. It has been observed that (7, 8) low FPG may increase the risk of osteoporosis in Chinese non-diabetic elderly women. Therefore, it is difficult to apply the association between diabetic patients and osteoporosis directly to non-diabetic patients. From reviewing the existing studies, there are no relevant studies in non-diabetic populations. The present study is intended to explore the relationship between MHR and osteoporosis in non-diabetic elderly populations.

## 2 Information and methods

### 2.1 Objects

Subjects who visited Guangdong Provincial Hospital of Chinese Medicine and underwent physical examination and completed bone mineral density examination from January 2020 to December 2023 were selected. A total of 240 subjects were recruited as the study population after screening for inclusion criteria and exclusion criteria.

#### 2.1.1 Inclusion criteria

(i) aged ≥50; (ii) no history of diabetes; (iii) complete clinical data;.

#### 2.1.2 Exclusion criteria

① treated by long-term drugs uses affecting glycolipid metabolism, (e.g. glucocorticoids, etc.); ② existence of other endocrine diseases affecting bone metabolism (e.g. thyroid disease, parathyroid disease, gonadal disease); ③ diagnosis of infectious states or blood diseases, etc.; ④the history of tumor-related diseases, such as multiple myeloma, thyroid cancer, etc.; ⑤ with serious abnormalities of liver and kidney functions;.

#### 2.1.3 Diagnostic criteria for osteoporosis

Referring to the World Health Organization’s recommended standard, the diagnostic criteria for osteoporosis are as follow: T-value ≥ -1.0 SD measured by dual-energy X-ray absorptiometry is considered normal bone mass, -2.5 SD < T-value < 1.0 SD is considered reduced bone mass, and T-value ≤ -2.5 SD is considered osteoporosis.

### 2.2 Methodology

Dual-energy X-ray bone mineral density and biochemical index results of 240 subjects were retrospectively detected. All biochemical indicators were measured at the Guangdong Provincial Hospital of Chinese Medicine and quality was controlled by the Laboratory Department. The indices included routine blood tests(including neutrophils(NEUT), lymphocytes(LYM), monocytes(MONO), red blood cell(RBC), hemoglobin(Hb), blood platelet(PLT)), fasting blood glucose (FPG), glycosylated hemoglobin (HbA1c), blood lipids (including triglycerides(TG), triglyceride(TC), high-density lipoprotein cholesterol(HDL-C), low-density lipoprotein cholesterol(LDL-C), non-high-density lipoprotein cholesterol(nonHDL-C),uric acid (UA), creatinine (Cr); MHR was calculated as the ratio of monocytes to high-density lipoprotein cholesterol.

### 2.3 Statistical analyses

SPSS 26.0 statistical software was used for the statistical analyses, with α= 0.05 as the test level of significance and p<0.05 considered statistically significant. For normally distributed continuous measures one-way ANOVA was used, and for non-normally distributed continuous measures non-parametric tests were used, all expressed as ( $\bar{x}\pm s$ ). Binary logistic regression was used to analyze the correlation between MHR and osteoporosis in the non-diabetic elderly population, and subject work characteristic (ROC) curves were plotted and the lower part of the curves and the critical values were calculated. Pearson’s test was used for the correlation analysis of continuous measures obeying normal distribution, and Spearman’s test was used for the correlation analysis of continuous measures not obeying normal distribution.

## 3 Results

**Figure 1:**
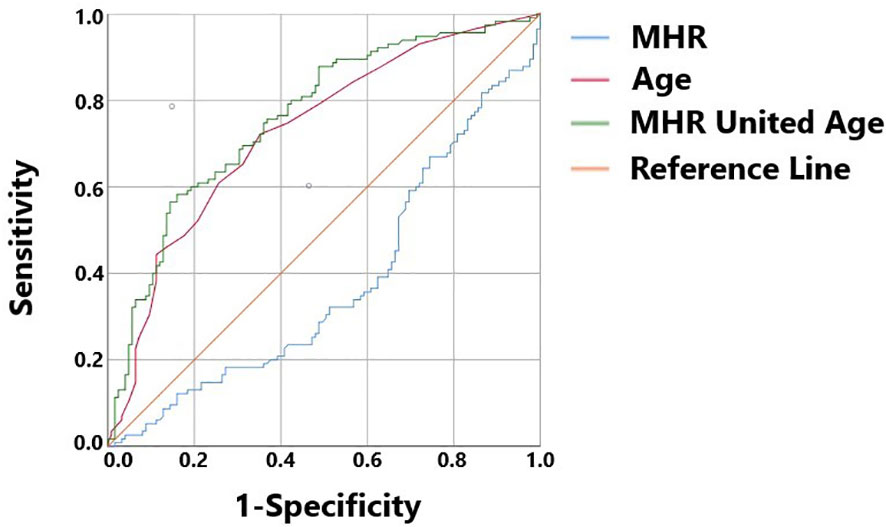
MHR, Age, and MHR combined with Age as ROC curves for the development of osteoporosis in the non-diabetic middle-aged and elderly populations.

**Figure 2:**
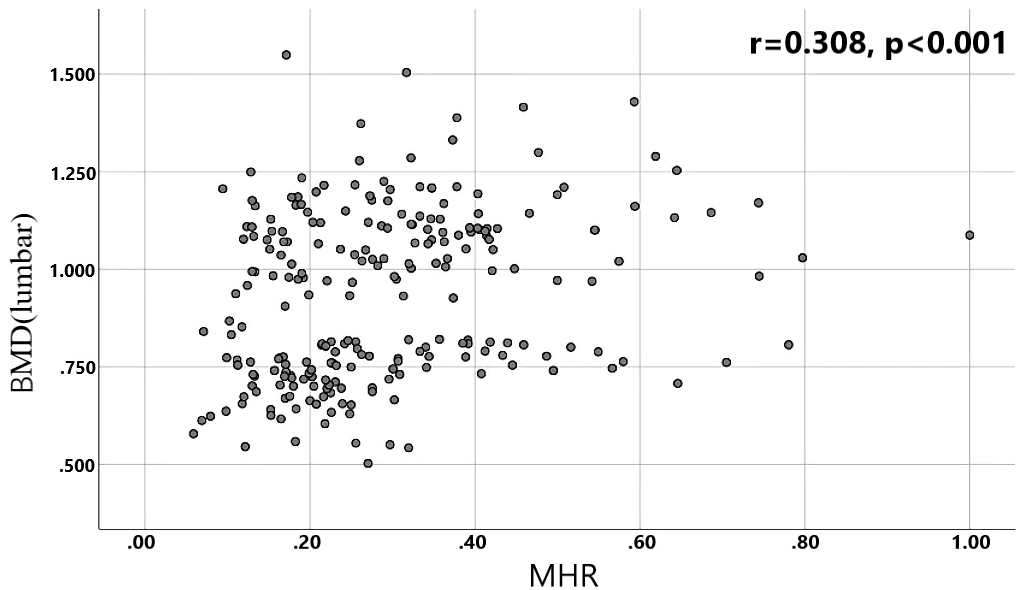
Correlation analysis of MHR with lumbar BMD.

**Figure 3:**
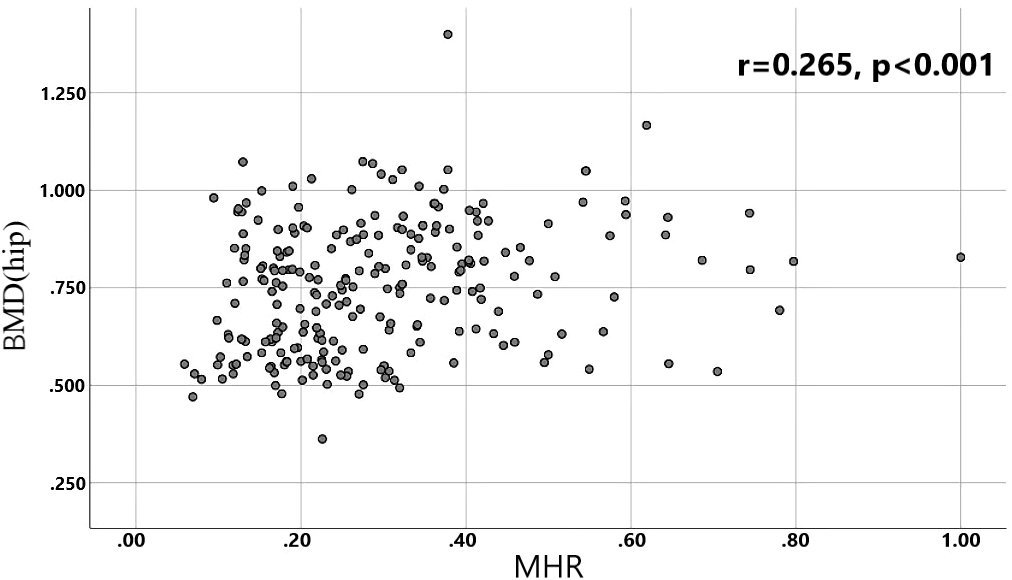
Correlation analysis of MHR with hip BMD.

**Figure 4:**
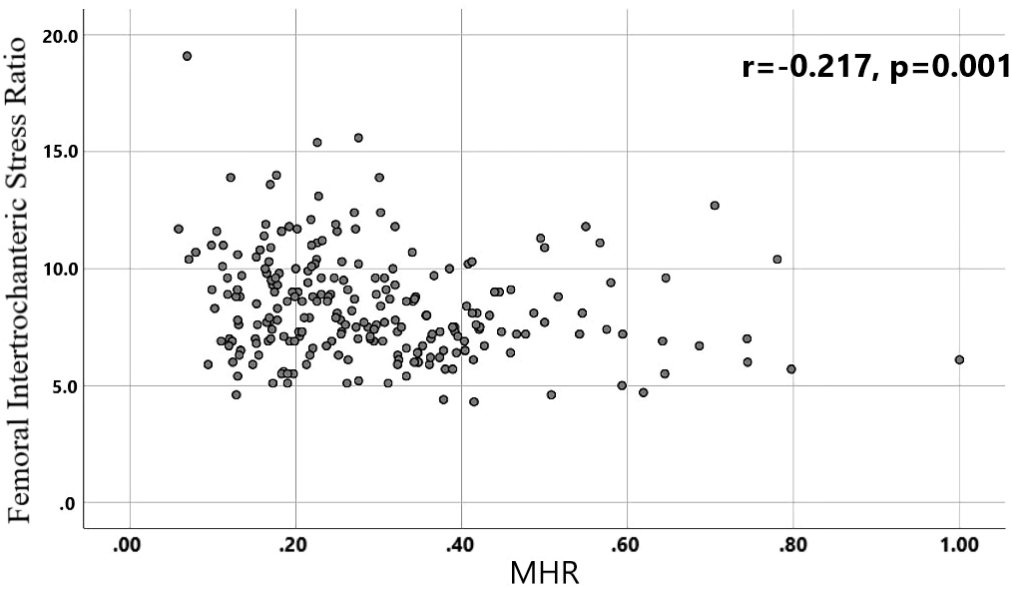
Correlation analysis of MHR with femoral intertrochanteric stress ratios.

**Figure 5:**
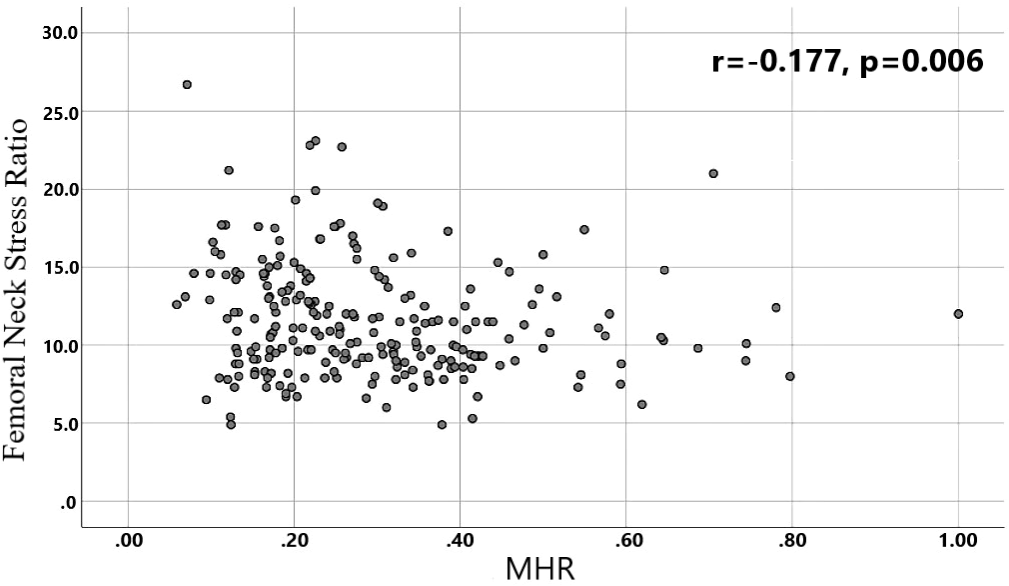
Correlation analysis of MHR with femoral neck stress ratios.

**Figure 6:**
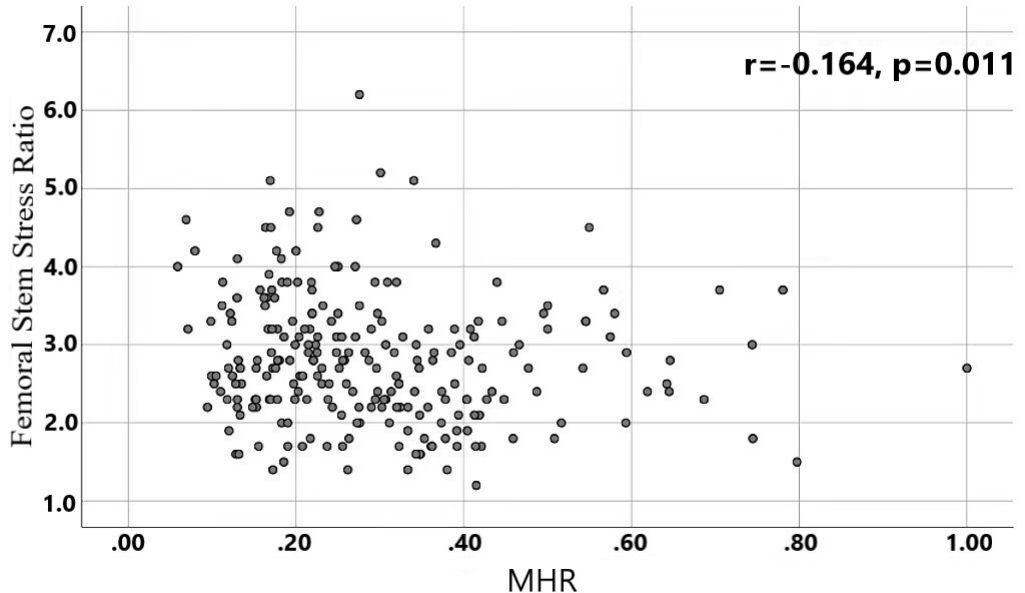
Correlation analysis of MHR with femoral stem stress ratios.

A total of 240 non-diabetic elderly populations were included, including 115 in the osteoporosis group and 125 in the normal bone group. From comparison of the general data the results showed that there were statistically significant differences in age, NEUT, LYM, MONO, MHR, UA, Cr, TG, and HDL-C (see **Tables 1**, **2**). The idea that chronic inflammation and glycolipid metabolism plays an important role in the development of osteoporosis is gradually being accepted. It has been reported that age (9), monocytes (10), HDL-C (11), UA (12), Cr (13) and independent predictors of osteoporosis. Therefore, we paid more attention to the study of these indicators of osteoporosis in non-diabetic patients. By conducting a binary logistic regression model and including age, MHR, UA, and Cr as variables (see **Table 3**), we found that age was an independent risk factor for bone abnormalities in non-diabetic elderly populations, and MHR was an independent protective factor for bone abnormalities in non-diabetic elderly populations. The ROC analysis showed that the area under the curve for the predictive effect of MHR, age and their combined test on osteoporosis in non-diabetic elderly populations was 0.623, 0.728 and 0.761, respectively; MHR alone has a high diagnostic value for bone abnormalities in non-diabetic elderly populations. The area under the receiver operating characteristic (ROC) curve was used to analyze the predictive accuracy of MHR. The optimal MHR cut-off value was determined using Youden’s index (14). The cutoff value with the highest Youden index (0.259) was defined as the optimization. The optimal value of MHR as a predictor of the development of osteoporosis was 0.259× 10^9^/mmol, which yielded a sensitivity of 62.4% and a specificity of 63.5%. MHR combined with age is a more suitable marker of bone abnormality for non-diabetic elderly populations, and the maximum value of the Youden index was calculated to be 0.423, corresponded to a sensitivity and specificity of 58.3% and 84.0%, respectively (see **Figure 1**); the correlation analysis showed that the MHR had a positive correlation with lumbar and hip BMD, and a negative correlation with the femoral neck stress ratio, the femoral intertrochanteric stress ratio, and the femoral stem stress ratio, with statistically significant differences (P<0.05). (See **Figures 2**–**6**).

**Table 1:** Comparison of general information in non-diabetic middle-aged and elderly populations.

	Osteoporosis group (n=115)	Normal bone mass group (n=125)	P
Age	60.90 ± 6.71	55.85 ± 5.81	<0.001
NEUT (10^9^/L)	3.31 ± 1.20	3.60 ± 1.34	0.018
LYM (10^9^/L)	1.91 ± 0.59	2.07 ± 0.60	0.044
MONO (10^9^/L)	0.35 ± 0.14	0.40 ± 0.14	0.001
RBC (10^9^/L)	4.78 ± 0.57	4.87 ± 0.57	0.292
Hb (10^9^/L)	141.70 ± 15.34	144.54 ± 14.83	0.147
PLT (10^9^/L)	243.49 ± 49.43	250.84 ± 56.10	0.284
MHR	0.26 ± 0.14	0.32 ± 0.16	0.001
HbA1c (%)	5.68 ± 0.34	5.61 ± 0.40	0.143
FPG (mmol/L)	5.32 ± 0.52	5.31 ± 0.53	0.699
UA (μmol/L)	352.95 ± 92.84	379.94 ± 84.47	0.019
Cr (μmol/L)	71.17 ± 15.89	75.89 ± 17.42	0.035
TG (mmol/L)	1.45 ± 1.23	1.59 ± 1.30	0.093
TC (mmol/L)	5.37 ± 1.05	5.34 ± 0.97	0.821
HDL-C (mmol/L)	1.47 ± 0.39	1.37 ± 0.35	0.030
nonHDL-C (mmol/L)	3.90 ± 1.02	3.96 ± 0.95	0.564
LDL-C (mmol/L)	3.52 ± 0.98	3.53 ± 0.92	0.961

**Table 2:** Bone density results between the two groups.

	Osteoporosis group (n=115)	Normal bone mass group (n=125)	P
Lumbar BMD	0.738 ± 0.09	1.112 ± 0.12	P<0.01
Lumbar T-score	-3.00 ± 0.83	0.39 ± 1.02	P<0.01
Hip BMD	0.613 ± 0.09	0.879 ± 0.10	P<0.01
Hip T-score	-2.27 ± 0.69	-0.23 ± 0.78	P<0.01
Femoral neck stress ratio	14.05 ± 3.40	9.25 ± 1.84	P<0.01
Femoral intertrochanteric stress ratio	10.04 ± 1.96	6.84 ± 1.12	P<0.01
Femoral stem stress ratio	3.23 ± 0.83	2.42 ± 0.61	P<0.01

**Table 3:** Independent predictors of the occurrence of osteoporosis in the non-diabetic middle-aged and elderly population analyzed by binary logistic regression.

	B	Wald X2	OR	95% CL	P
Age	0.137	31.503	1.147	1.094~1.204	<0.001
MHR	-2.679	5.018	0.069	0.007~0.715	0.025
UA	-0.002	1.058	0.998	0.994~1.002	0.304
Cr	-0.006	0.352	0.994	0.974~1.014	0.553

## 4 Discussion

Age-related increases in oxidative stress, osteoclastogenesis and bone loss are important causes of osteoporosis in the elderly (15). The immune and skeletal systems share multiple regulatory factors, and postmenopausal osteoporosis is suggested (16) to be associated with dysfunction of T and B lymphocytes; whereas monocytes are innate immune cells of the myeloid lineage derived from bone marrow and play an important role in tissue homeostasis and immune responses (17). Under the regulation of various factors, monocytes can fuse to form multinucleated osteoclasts. D’Amelio, P (18), et al. collected data on bone mineral density, markers of bone turnover, and cultures of monocytes on dentine slices from 18 osteoporotic women and 15 controls. They found a significant increase in osteoclast formation and bone resorption activity in patients compared to the controls. This demonstrated an increased spontaneous osteoclastogenesis in women affected by postmenopausal osteoporosis. While in the presence of decreased endogenous antioxidant production (19), excessive accumulation of endogenous glucocorticoids (17) and other specific conditions, it may increase the level of monocytes in the blood and bone marrow, and even enhance the osteoclastogenic potential and inflammatory capacity of monocytes, thereby leading to the development of bone diseases. Therefore, monocytes may play an important role in the pathological mechanism underlying OP progression.

Osteoblasts, chondrocytes and adipocytes all originate from mesenchymal stem cells, and when the signaling pathway is altered, bone marrow stromal cells tend to differentiate into adipocytes, thus, it constitutes the physiological basis for the close relationship between lipid metabolism and bone metabolism. It has been found that osteoporosis may be related to serum cholesterol (20) levels, and that individuals with osteoporosis and bone mass loss usually have abnormal lipid metabolism, especially serum HDL (3). Individuals with osteoporosis and bone mass loss often exhibit abnormalities in lipid metabolism, particularly in serum HDL-C, which reduces bone mineral density by stimulating a decrease in osteoblast number and function (11). Sun, Yongbing (3) found that elevated HDL-C levels affect bone loss in Chinese people aged 20 to 80 years, and the decline in BMD is particularly prominent in obese men; however, bone metabolism and lipid metabolism do not have a simple linear relationship across subgroups of age, gender, and ethnicity. Xie, Ruijie (21) et al. collected and analyzed data from the 2011-2018 National Health and Nutrition Examination Survey (NHANES) and found a positive correlation between HDL-C and lumbar BMD in 20- to 59-year-olds (n = 10,635), which is inconsistent with previous studies. Dule, Sara (22) also assert in their study that low levels of HDL-C are significantly and independently associated with bone deterioration in women with T2DM. Studies have shown that (11) HDL-C decreases bone mineral density by decreasing the number and function of osteoblasts, and that high levels of HDL-C may be implicated in the occurrence of fracture events in the elderly. Therefore, the relationship between HDL-C and osteoporosis is complicated, and the underlying mechanisms need to be further investigated.

It has been concluded that high-density lipoprotein (HDL) is an anti-inflammatory factor that can inhibit the expression of inflammatory factors by preventing p38 MAP kinase activation (23), etc. Murphy (24) et al. have also found that HDL can effectively inhibit the expression of integrin CD11b on the surface of monocytes. HDL-C (25) protects the endothelium or blood vessels from inflammation and oxidative stress by preventing LDL-C oxidation and the entry of monocytes into the vessel wall. Even the inhibitory effect of HDL on monocytes was effective in both preventive and reversal situations, and experimental studies found that monocytes in the prestimulated state could be rescued from activation by HDL. The combination of monocytes and HDL to form a new integrated inflammatory indicator, MHR, that incorporates both immune and protective mechanisms may have greater clinical value than single monocyte and HDL levels.

MHR is a defined indicator of systemic inflammation and abnormal lipid metabolism. Many studies have proven its association with cardiovascular disease (26), diabetic complications (27) and psychiatric disorders (28). However, the relationship between MHR and osteoporosis remains unclear. ZHU Guang (29) et al. studied 227 elderly populations with T2DM and found that MHR was an independent risk factor for osteoporosis. The MHR in the osteoporosis group was significantly higher than in the normal group and the bone loss group (P<0.05); however, the results of the present study showed that MHR was a protective factor for nondiabetic patients over the age of 50 years, and that the MHR in the normal bone group was significantly higher than in the osteoporosis group, which may be related to the different metabolic conditions in the body of non-diabetic and diabetic patients.

Since bone density screening is not fully popularized in the clinic, many middle-aged and elderly people are diagnosed with osteoporosis only after fracture, resulting in poor disease prognoses. Therefore, we try to discover novel biomarkers that can be obtained reliably, economically, and easily accessible and used in the prediction of clinical disease, which may help in the early detection of disease and improve the prognosis of osteoporosis.

Based on our study, we believe that MHR is a good indicator. However, the intrinsic mechanism between MHR and osteoporosis is complicated, and its significance may not be consistent in different population subgroups, which needs to be explored in further studies.

## Author contributions

XL: Writing – review & editing, Writing – original draft. MY: Writing – review & editing, Writing – original draft, Data curation. JJ: Writing – original draft, Data curation. ZM: Writing – original draft.
